# Effect of Catalytic Pyrolysis Conditions Using Pulse Current Heating Method on Pyrolysis Products of Wood Biomass

**DOI:** 10.1155/2014/720527

**Published:** 2014-12-31

**Authors:** Sensho Honma, Toshimitsu Hata, Takashi Watanabe

**Affiliations:** ^1^Hokkaido Research Organization, Forest Products Research Institute, 1-10 Nisikagura, Asahikawa, Hokkaido 071-0198, Japan; ^2^Kyoto University Research Institute for Sustainable Humanosphere, Gokanosho, Uji, Kyoto 611-0011, Japan

## Abstract

The influence of catalysts on the compositions of char and pyrolysis oil obtained by pyrolysis of wood biomass with pulse current heating was studied. The effects of catalysts on product compositions were analyzed using GC-MS and TEM. The compositions of some aromatic compounds changed noticeably when using a metal oxide species as the catalyst. The coexistence or dissolution of amorphous carbon and iron oxide was observed in char pyrolyzed at 800°C with Fe_3_O_4_. Pyrolysis oil compositions changed remarkably when formed in the presence of a catalyst compared to that obtained from the uncatalyzed pyrolysis of wood meal. We observed a tendency toward an increase in the ratio of polyaromatic hydrocarbons in the pyrolysis oil composition after catalytic pyrolysis at 800°C. Pyrolysis of biomass using pulse current heating and an adequate amount of catalyst is expected to yield a higher content of specific polyaromatic compounds.

## 1. Introduction

Fossil fuels are currently the world's most dominant energy source. However, fossil fuels are nonrenewable sources of energy and their use leads to negative effects on the environment. These issues have led to an increased interest in renewable resources such as biomass. Biomass pyrolysis is the thermal degradation of lignocellulose in the absence of oxygen to form charcoal, pyrolysis oil, and gaseous fuel products. Depending on the operating conditions, biomass pyrolysis can be subdivided into slow and fast pyrolysis, of which the latter has been receiving a lot of attention as it maximizes pyrolysis oil production [1, 2]. One of the most important methods to improve the quality of pyrolysis oil is the addition of heterogeneous catalysts during biomass pyrolysis [3].

Pyrolysis employing the pulse current heating method can be applied to catalyzed pyrolysis technology for the production of useful substances from wood biomass [4–10]. A judicious choice of catalysts and the use of optimal pyrolysis conditions make it possible to produce more useful chemical feedstocks in a selective manner. The applicability of pyrolysis oil in fuels, chemicals, and functional materials has been proposed in previous studies describing catalytic pyrolysis processes [11, 12]. Torri et al. reported the effects on the carbon yield of products in the pyrolysis of pine sawdust at 500°C, using 31 types of catalysts, including zeolite ZSM-5, TiO_2_, and Fe_2_O_3_ [13]. Lu et al. reported the effects of six different metal oxides, including TiO_2_ and Fe_2_O_3_, on the composition of pyrolysis oil using Py-GC/MS [14]. On the other hand, only a few studies have been conducted on the effects on the liquefaction in pyrolysis of wooden biomass at 800°C using titanium oxide and iron oxide, while investigations about the effects of catalysts on the composition of the oil from the pyrolysis at 500–600°C have been reported. The selective production of useful substances can be achieved by modifying and improving the properties of pyrolysis oil as a consequence of the synthesis conditions employed. An increase in the composition ratio of aromatic hydrocarbons in pyrolysis oil was reported [15, 16] when zeolite and some metal oxides were present in biomass during the pyrolysis process. Furthermore, we expect a reduction in the energy required for the production of pyrolysis oil if fuel or functional materials can be produced from pyrolysis oil at lower temperatures with the aid of catalysts.

Catalytic graphitization is one chemical process from which the physical properties of char formed during pyrolysis originate. Partial graphitization is known to occur during pyrolysis of organic matters when conducted in the presence of iron oxide, titanium oxide, nickel compounds, or other materials. The following two mechanisms are recognized to operate in catalytic graphitization [17]: (1) dissolution-precipitation and (2) carbide formation. Low-temperature graphitization occurs in graphitizable carbon; the efficient catalytic action of transition-metal elements and their oxides affects the crystallization of carbon [18, 19]. Similar effects are expected in pyrolysis using the pulse current heating method. The effects of the catalytic reactions employing titanium oxide or iron oxide, which have different graphitization mechanisms, on the microtexture of char, produced from wood biomass pyrolysis at 800°C by using a pulse current heating method, are of interest in understanding the process of microstructural change in wood during fast pyrolysis.

Some reports have characterized the char obtained from woody biomass/metal-oxide mixtures by pyrolysis with a pulse current heating system [4, 20]. On the other hand, the benefits like efficient production, useful chemicals, and functional char are expected by production of both pyrolysis oil and char in one step and the composition of pyrolysis oil produced by the pulse current heating system. Characterization of char and pyrolysis oil, obtained from pyrolysis by the pulse current heating method, is necessary to clarify the influence of catalytic conditions on product composition. We also determined product distributions at a given processing temperature. In this study, Japanese cedar wood meal was used for pyrolysis since its raw material waste can be obtained easily. Japanese cedar is one of the most important species in Japan for use as forest products and wood biomass [21], as described previously [10]. The catalysts iron(II, III) oxide (Fe_3_O_4_), titanium(IV) oxide (TiO_2_), and zeolite ZSM-5, which have different mechanisms of graphitization, were examined. We also analyzed the chemical components of liquefaction and characterized the microtexture of the pyrolysis residue obtained at 500 and 800°C using these catalysts.

## 2. Materials and Methods

### 2.1. Raw Material and Catalysts

**Figure 1:**
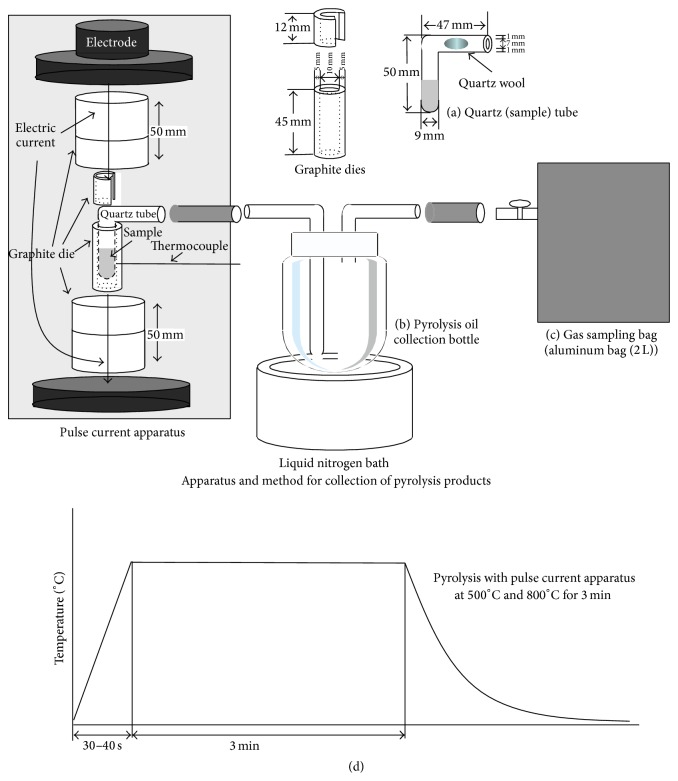
Apparatus and method for collection of pyrolysis products: (a) graphite dies and quartz tube with sample, (b) pyrolysis oil in collection bottle, (c) gas sampling bag (aluminum bag (2 L)), and (d) Heating pattern used for pyrolysis using pulse current apparatus.

Sapwood meal ground by a ball mill from Japanese cedar (*Cryptomeria japonica*) wood from Wakayama prefecture with age of 30 was used as a raw material. Fe_3_O_4_ (powder, Wako Pure Chemical Industries, Ltd.), TiO_2_ (anatase-type powder, Kishida Chemical Co., Ltd.), and zeolite ZSM-5 (Zeolyst International, CVB 3024E, nominal cation form: ammonium) were used as catalysts. Wood meal and catalyst powder were mixed in a 1 : 1 (w/w) ratio. The apparatus used for pyrolysis by pulse current heating incorporates a graphite mold in which a quartz tube containing the powdered sample (Figure 1(a)) is inserted and heated by conducting an electric current through the graphite mold [4–7, 10]. For comparison, a woody powder sample (100 mg) was pyrolyzed under the heating conditions as shown in Figure 1.

### 2.2. Pyrolysis with Pulse Current Heating Method

The sample powder in the apparatus (Figure 1) was pyrolyzed by electric heating. Pyrolysis experiments were conducted at temperature of 500°C and 800°C, the pyrolysis time of 3 min, and the heating rate of 15–20°C/s. The choice of heating rate and pyrolysis time was based on a previous report by Carlson et al. [16]. The heating pattern used for the pyrolysis experiments is shown in Figure 1(d). The heating rate was set to 15–20°C/s in order to obtain both pyrolysis oil and char effectively. The current was raised to 800–950 A at 14–16 V for a few seconds to realize a heating rate of 15–20°C/s and then decreased to 300–450 A at 3–4.5 V to maintain the sample at the required pyrolysis temperature. The quartz tube, collection bottle, and gas-sampling bag were arranged in a closed system. An L-shaped quartz tube (Figure 1(a)) helped to lead the volatiles produced by pyrolysis to the collection bottle without disturbing the electric current. The liquid fraction containing volatilized pyrolysis oil components formed during pyrolysis was cooled with liquid nitrogen for recovery and collected in the washing tube, as shown in Figure 1(b). The volatiles trapped inside the quartz tube and connector were washed with acetone and then analyzed quantitatively and qualitatively as pyrolysis oil. The solid fraction remaining inside the quartz tube was also collected by washing and filtration. The amount of char was quantified by obtaining the weight of the dried sample.

### 2.3. TEM Analysis of Char

The chars obtained by catalytic pyrolysis were analyzed using TEM (JEOL 2010F) with electron energy-loss spectroscopy (EELS) (Gatan Enfina). Selected area electron diffraction (SAED) was taken in addition to imaging.

### 2.4. GC-MS Analysis of Pyrolysis Oil

GC-MS analyses and semiquantification of pyrolysis oil chemical components were performed on a QP-5050A GC-MS (Shimadzu Co.). Tetracosane (C_24_H_50_) was used as an internal standard. A DB-5 capillary column was used (30 m × 0.25 mm in diameter, film thickness of 1.0 *μ*m, Agilent Co.) and a splitless mode was used for injection, with an injector temperature of 280°C. The oven temperature was programmed to rise from 30°C (5 min hold) to 240°C at a rate of 14°C/min and from 240°C to 310°C (18 min hold) at a rate of 30°C/min. High-purity helium was used as the carrier gas at a flow rate of 40.3 mL/min. The components of pyrolysis oil were determined from the obtained total ion chromatogram (TIC) using the Kovats retention index method [22–30] and a mass spectral library. Peak areas from TIC plots were examined for semiquantification.

## 3. Results and Discussion

### 3.1. Product Distribution

**Figure 2:**
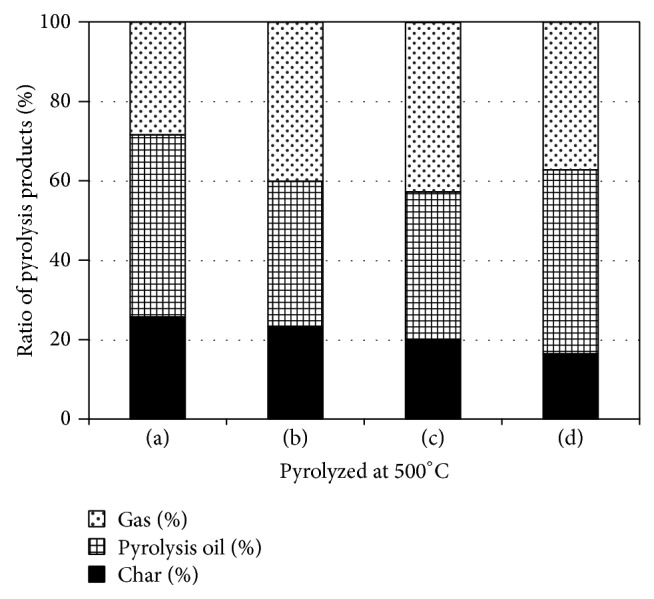
Product distribution of pyrolysis products obtained from Japanese cedar wood meal/catalyst (1 : 1 w/w) mixtures pyrolyzed at 500°C: (a) Japanese cedar wood meal [10], (b) Wood meal/Fe_3_O_4_ mixture, (c) Wood meal/TiO_2_ mixture, and (d) Wood meal/ZSM-5 mixture.

**Figure 3:**
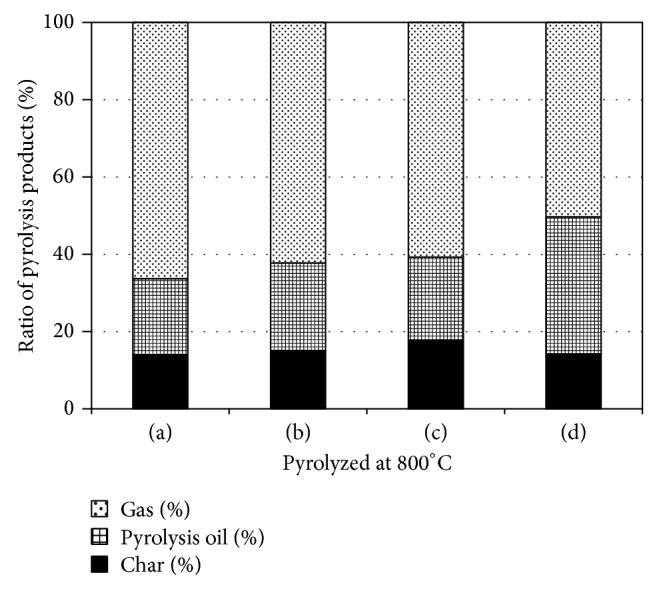
Product distribution of pyrolysis products obtained from Japanese cedar wood meal/catalyst (1 : 1 w/w) mixtures pyrolyzed at 800°C: (a) Japanese cedar wood meal [10], (b) Wood meal/Fe_3_O_4_ mixture, (c) Wood meal/TiO_2_ mixture, and (d) Wood meal/ZSM-5 mixture.

First, we examined the effects of catalyst addition on product distribution during pyrolysis by the pulse current heating method. The changes in composition ratios of the products obtained from wood meal are shown in Figures 2 and 3. The differences of the yield of pyrolysis products were not so remarkable among pyrolysis conditions with and without catalyst. Pyrolysis oil and char yields of 37–46% and 16–23%, respectively, were obtained at a processing temperature of 500°C, although both of these yields decreased with the addition of catalyst. Pyrolysis oil and char yields of 22–35% and 14–18%, respectively, were obtained at 800°C. Similar yields of products were obtained by catalyst addition in this experiment and almost no decrease in the yields of pyrolysis oil and char was observed.

The effects of catalyst addition on product distribution were investigated by comparing the results obtained in this case to those obtained in the absence of catalysts. The reaction time and addition of catalysts such as ZSM-5, titanium oxide, and iron oxide had little influence on product distribution. At 800°C, the yield of pyrolysis oil and char with catalyst addition was independent of the type of catalyst except that the yield of pyrolysis oil increased slightly when ZSM-5 was used as the catalyst. Possible reasons for this include the following: (i) the secondary reaction of pyrolysis oil and char did not occur on catalyst addition at 800°C, (ii) the ratio of catalyst to wood meal was 1 : 1, and (iii) the amount of added catalyst was not as high as that used by Carlson et al. [16], who reported that the carbon yield of oxygenated species decreased and that of hydrocarbon, CO, and CO_2_ gas increased with the catalyst (ZSM5) to glucose ratio from 9 : 1. The yield of pyrolysis oil was considered to be kept at the same level as oxygenated species decreased while aromatic hydrocarbons increased. The effects of catalyst addition on the decomposition of oxygenated compounds and polycyclic-aromatic-hydrocarbon production are already known [31, 32]. A similar tendency was observed for pyrolysis conducted at both 500 and 800°C. Consequently, the addition of catalysts had little effect on the char yield. The influence on physical properties by the char and pyrolysis oil composition will be mentioned in Sections 3.2 and 3.3.

### 3.2. TEM Analysis of Char

**Figure 4:**
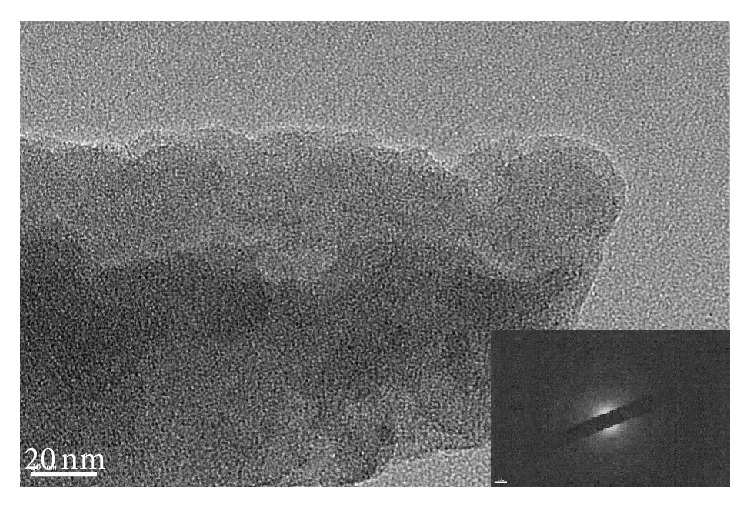
TEM image and SAED of char obtained from Japanese cedar wood meal pyrolyzed at 800°C.

**Figure 5:**
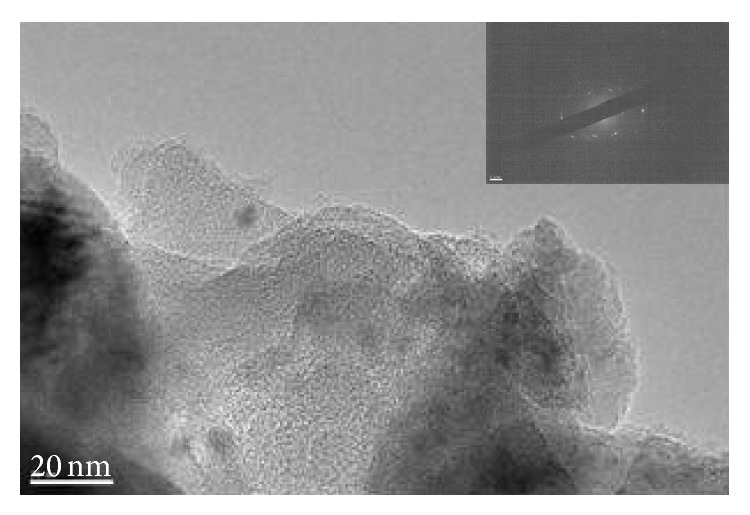
TEM image and SAED of char obtained from mixture of Japanese cedar wood meal/Fe_3_O_4_ (1 : 1 w/w) pyrolyzed at 800°C.

**Figure 6:**
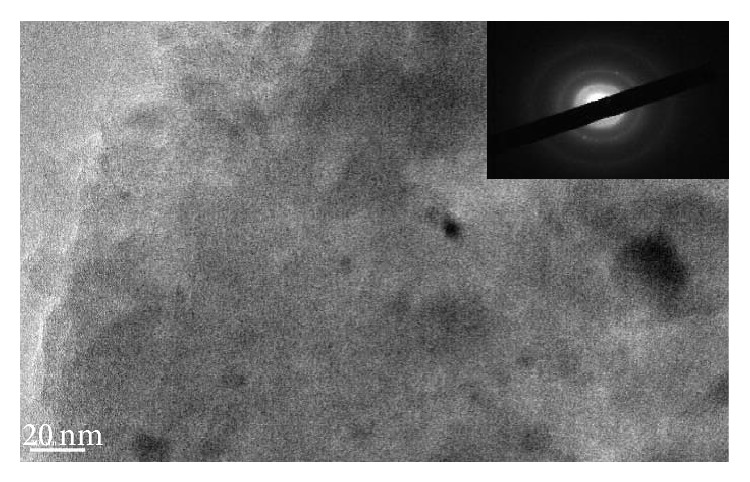
TEM image and SAED of char obtained from mixture of Japanese cedar wood meal/TiO_2_ (1 : 1 w/w) pyrolyzed at 800°C.

**Figure 7:**
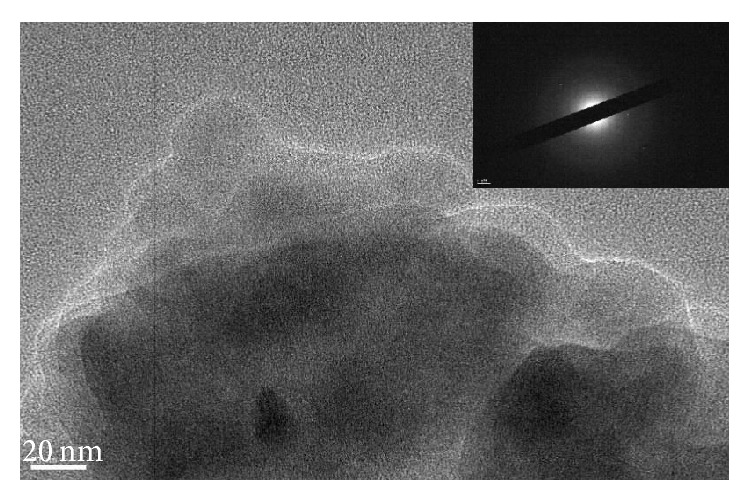
TEM image and SAED of char obtained from mixture of Japanese cedar wood meal/zeolite (ZSM-5) (1 : 1 w/w) pyrolyzed at 800°C.

Pyrolysis residues obtained at 800°C were examined with TEM and SAED. The results of analysis of wood meal, wood meal/Fe_3_O_4_ (1 : 1 w/w), wood meal/TiO_2_ (1 : 1 w/w), and wood meal/ZSM-5 (1 : 1  w/w) pyrolyzed at 800°C are shown in Figures 4, 5, 6, and 7, respectively. TEM and SAED analysis indicated the coexistence or dissolution of amorphous carbon and iron oxide in the wood meal/Fe_3_O_4_ sample pyrolyzed at 800°C in Figure 5, which were not observed in the pyrolysis residue of Japanese cedar wood meal pyrolyzed without catalyst at 800°C in Figure 4. From these results, state of pyrolysis residue of the wood meal/Fe_3_O_4_ sample carbonized at 800°C was considered to be in relation to a process of catalytic graphitization. Consequently, as expected, the pyrolysis with pulse current heating process conducted at 800°C with Fe_3_O_4_ addition contributed to changes of char in physical properties. The phenomenon of coexistence or dissolution was more evident at the interface of carbon and oxides in the case of added Fe_3_O_4_ shown in Figure 5 than in the case of added TiO_2_ in Figure 6.

### 3.3. Composition of Pyrolysis Oil

**Figure 8:**
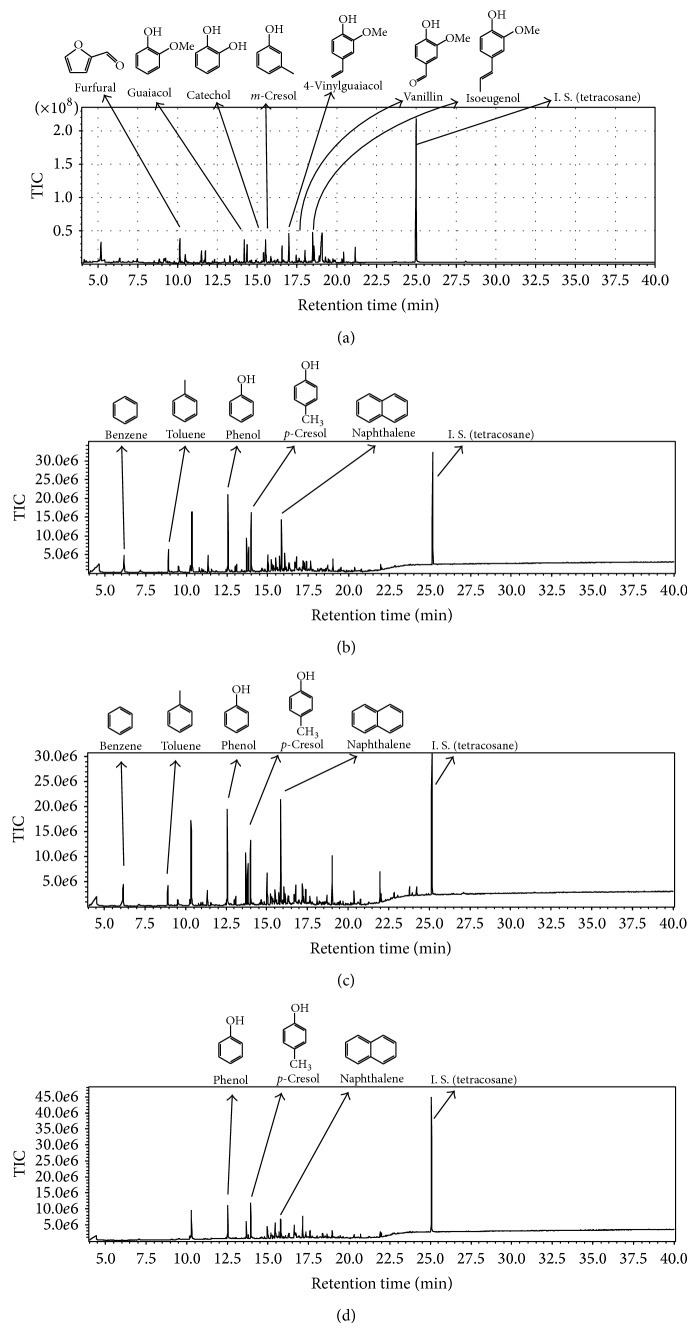
TIC of pyrolysis oils obtained from of Japanese cedar wood meal/catalyst (1 : 1 w/w) mixtures pyrolyzed at 500°C: (a) without catalyst [10], (b) Fe_3_O_4_, (c) TiO_2_, and (d) ZSM-5.

The composition of pyrolysis oil was found to change drastically on catalyst addition compared to results obtained from wood meal pyrolysis in the absence of catalysts. More polyaromatic hydrocarbons (PAHs) were observed in the pyrolysis oil of the Japanese cedar sample at 500°C with the three kinds of catalyst. The composition ratios of specific aromatic hydrocarbons, including naphthalene, tended to increase at 800°C. Compounds with oxygen functional groups, such as isoeugenol, vanillin, furfural, and 4-vinylguaiacol, were previously reported to be present in the pyrolysis oil obtained from wood meal pyrolyzed without a catalyst at 500°C [10]. Aromatic hydrocarbons (benzene, toluene, naphthalene, etc.) and phenols (phenol, cresol) were identified in the pyrolysis oil obtained from both wood meal/Fe_3_O_4_ (1 : 1 w/w) and wood meal/TiO_2_ (1 : 1 w/w) samples pyrolyzed at 500°C, while the ratio of compounds with oxygen functional groups decreased relatively (Table 1 and Figure 8). A similar tendency was shown in the pyrolysis oil from the wood meal/ZSM-5 (1 : 1  w/w) sample pyrolyzed at 500°C (Table 1 and Figure 8), although the results were not as remarkable as those for the wood meal/Fe_3_O_4_ and wood meal/TiO_2_ samples. From the above results, catalyst addition during pyrolysis at 500°C was considered to be effective in increasing aromatic hydrocarbon content in the resulting pyrolysis oil.

Many kinds of aromatic compounds with oxygen functional groups were included in the pyrolysis oil obtained from wood biomass by pyrolysis at 500°C, and the amounts of aromatic hydrocarbon or polyaromatic compounds increased at 700 to 800°C from previous reports [12, 33]. Each catalyst was considered to be effective in obtaining pyrolysis oil with similar compositions when conducting pyrolysis at higher temperatures, and the yield of each pyrolysis oil did not remarkably decrease; consequently, an increase in aromatic hydrocarbon yield and reduction in pyrolysis energy were achieved. These results are in good agreement with previous reports on the increase in aromatic hydrocarbon yields under pyrolysis conditions using zeolite catalysts [16, 34].

**Figure 9:**
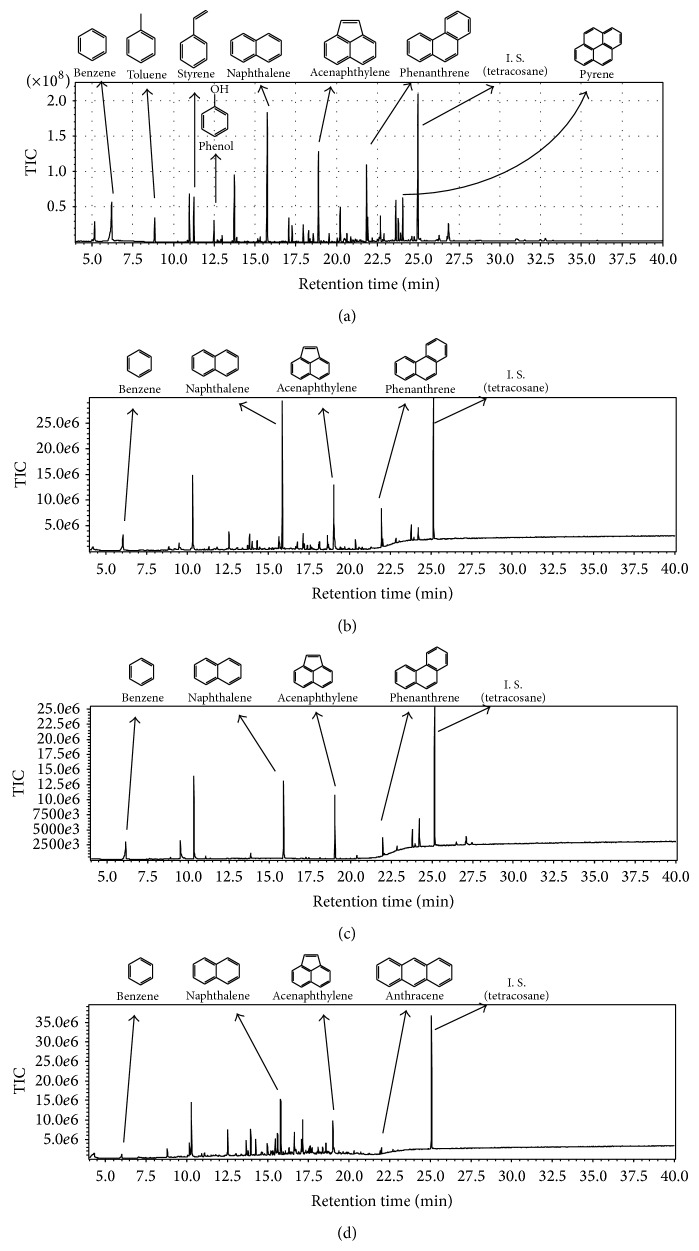
TIC of pyrolysis oils obtained from of Japanese cedar wood meal/catalyst (1 : 1 w/w) mixtures pyrolyzed at 800°C: (a) without catalyst [10], (b) Fe_3_O_4_, (c) TiO_2_, and (d) ZSM-5.

The weight ratios of many components decreased in pyrolysis oil obtained from both wood meal/Fe_3_O_4_ (1 : 1 w/w) and wood meal/TiO_2_ (1 : 1 w/w) samples pyrolyzed at 800°C, whereas the weight ratio of some PAHs (such as naphthalene) increased (Table 2, Figure 9). Aromatic hydrocarbons such as benzene, toluene, and naphthalene were mainly detected in pyrolysis oil obtained from wood meal pyrolyzed in the absence of catalyst at 800°C [10]. Since similar yields of pyrolysis oil were obtained (Figures 2 and 3) in both catalyzed and uncatalyzed pyrolysis processes, the pyrolysis of biomass with pulse current heating using a suitable amount of catalyst was expected to generate specific aromatic compounds in higher ratios. Aromatic hydrocarbons such as benzene, toluene, and naphthalene were detected in the pyrolysis oil obtained from the wood meal/ZSM-5 (1 : 1 w/w) sample pyrolyzed at 500°C; phenol, cresol, catechol, and guaiacol were also detected. The weight ratio of PAHs increased in the pyrolysis oil obtained from wood meal/ZSM-5 sample pyrolyzed at 800°C, and pyrolysis oil composition showed a similar tendency to that obtained from wood meal/Fe_3_O_4_ and wood meal/TiO_2_ samples. From these results, pyrolysis using biomass with an adequate amount of catalyst is expected to yield specific aromatic compounds in a higher content, as a similar yield of pyrolysis oil was obtained from catalytic pyrolysis of the wood meal/ZSM-5 (1 : 1 w/w) sample at 800°C, compared to pyrolysis without catalyst.

In this research, the formation of aromatic hydrocarbons was observed during pyrolysis at 500°C, and the formation of PAHs, such as naphthalene and phenanthrene, could be increased by carrying out pyrolysis at 800°C in the presence of catalysts. Pyrolysis oil compositions were affected by the addition of catalysts such as iron oxides, titanium oxides, and zeolite ZSM-5. The promotion of PAH production is the catalytic effect of iron oxide at 600°C, the production of hydrocarbon increased slightly, and the effect of deoxygenating of pyrolysis vapor was low by using titanium oxide as reported by Lu et al. [14]. The catalytic effect of zeolite ZSM-5 on fast-pyrolysis oil compositions was reported for the decomposition of oxygenated compounds, producing aromatic hydrocarbon and polycyclic aromatic hydrocarbon, by Carlson et al. [16], Bridgwater [31], and Zhang et al. [32]. The effect of these catalysts on production of aromatic hydrocarbon and PAH promoted the decomposition of oxygenated constituents during pyrolysis under a pulse current heating. The amount of polycyclic aromatics especially increased with iron oxide addition.

## 4. Conclusion

The effects of catalytic conditions on pyrolysis of wood biomass in product distribution, as well as characterization of pyrolysis oil and the composition of char obtained simultaneously, were examined. Consequently, the composition of some aromatic compounds changed noticeably on using metal oxides as a catalyst. Furthermore, the phenomenon of coexistence or dissolution at the interface of the carbon and oxides was more remarkable in the case of Fe_3_O_4_ than in the case of TiO_2_. The pyrolysis oil composition ratio was changed remarkably on using a catalyst, compared to results obtained from wood meal pyrolysis in the absence of catalysts. The tendency toward an increase in the ratio of PAHs such as naphthalene in the pyrolysis oil was shown by catalytic pyrolysis with pulse current heating at 800°C. In this report, Fe_3_O_4_ was found to be more interesting as a catalyst for providing suitable pyrolysis oil composition and char organizational structure in pyrolysis of wood biomass by the pulse current heating method. An examination of the effect of iron oxide species, which differ in chemical composition and structure, on the characterization of pyrolysis products should be expected for further investigation.

## 5. Highlights

The following are the main highlights of the paper.

- The influence of catalysts on the compositions of char and pyrolysis oil obtained by pyrolysis of wood biomass with pulse current heating was studied.
- A tendency toward an increase in the ratio of polyaromatic hydrocarbons (such as naphthalene) in the pyrolysis oil composition via catalytic pyrolysis with pulse current heating at 800°C was shown.
- The coexistence or dissolution of amorphous carbon and iron oxide was observed in char pyrolyzed at 800°C with an Fe_3_O_4_ catalyst.

## Figures and Tables

**Table 1:** GC-MS semiquantitative analysis of pyrolysis oil components obtained from Japanese cedar wood meal/catalyst (1 : 1 w/w) mixtures pyrolyzed at 500°C.

Retention time	Pyrolysis oil	Pyrolysis oil	Pyrolysis oil	Pyrolysis oil
	Japanese cedar wood meal [10]	Mixture with Fe_3_O_4_	Mixture with TiO_2_	Mixture with ZSM-5
(min)	Area/I. S. area	Area/I. S. area	Area/I. S. area	Area/I. S. area
6.21 Benzene	—	0.32	0.27	—
8.85 Toluene	—	0.24	0.16	—
10.14 Furfural	0.12	0.11	0.07	0.04
10.97 Ethynylbenzene	—	0.03	—	—
11.25 Styrene	—	0.18	0.13	—
12.48 Phenol	—	0.73	0.64	0.28
13.62 *o-*Cresol	—	0.29	0.30	0.14
13.74 1-Propynylbenzene	—	—	—	—
13.90 *p-*Cresol	—	0.57	0.48	0.26
14.19 Guaiacol	0.11	0.04	—	—
15.40 Catechol	0.09	0.20	0.17	0.17
15.57 *m-*Cresol	0.16	0.04	—	—
15.77 Naphthalene	—	0.51	0.82	0.17
17.07 Methylnaphthalene	—	0.18	0.19	—
17.45 Eugenol	0.04	0.07	—	—
17.95 Biphenyl	—	—	0.06	—
18.00 Vanillin	0.08	0.04	—	—
18.49 Isoeugenol	0.12	0.05	0.04	—
18.89 Acenaphthylene	—	0.12	0.30	—
20.22 *o-*Biphenylenemethane	—	—	—	—
21.84 Phenanthrene	—	0.05	0.16	—
21.91 Anthracene	—	—	—	—
24.05 Pyrene	—	—	—	—
25.00 Internal standard (tetracosane)	1.00	1.00	1.00	1.00

**Table 2:** GC-MS semiquantitative analysis of pyrolysis oil components obtained from Japanese cedar wood meal/catalyst (1 : 1 w/w) mixtures pyrolyzed at 800°C.

Retention time	Pyrolysis oil	Pyrolysis oil	Pyrolysis oil	Pyrolysis oil
	Japanese cedar wood meal [10]	Mixture with Fe_3_O_4_	Mixture with TiO_2_	Mixture with ZSM-5
(min)	Area/I. S. area	Area/I. S. area	Area/I. S. area	Area/I. S. area
6.21 Benzene	0.63	0.21	0.25	0.04
8.85 Toluene	0.18	0.03	0.01	0.06
10.14 Furfural	—	—	—	—
10.97 Ethynylbenzene	0.26	—	0.02	—
11.25 Styrene	0.24	0.03	0.01	—
12.48 Phenol	0.12	0.14	—	0.28
13.62 *o-*Cresol	—	0.03	—	0.14
13.74 1-Propynylbenzene	0.50	—	—	—
13.90 *p-*Cresol	—	0.07	—	0.22
14.19 Guaiacol	—	0.07	—	0.19
15.40 Catechol	—	—	—	0.18
15.57 *m-*Cresol	—	—	—	—
15.77 Naphthalene	0.95	1.15	0.61	0.47
17.07 Methylnaphthalene	0.12	0.04	0.02	0.27
17.45 Eugenol	—	0.03	—	—
17.95 Biphenyl	0.10	—	0.01	0.09
18.00 Vanillin	—	0.07	—	—
18.49 Isoeugenol	—	0.11	—	—
18.89 Acenaphthylene	0.59	0.65	0.41	0.36
20.22 *o-*Biphenylenemethane	0.24	—	—	—
21.84 Phenanthrene	0.37	0.24	0.12	0.05
21.91 Anthracene	0.14	0.05	—	0.14
24.05 Pyrene	0.29	0.19	0.24	—
25.00 Internal standard (tetracosane)	1.00	1.00	1.00	1.00
